# Selenium foliar application alleviates salinity stress in sweet william (*Dianthus barbatus* L.) by enhancing growth and reducing oxidative damage

**DOI:** 10.1038/s41598-025-89463-6

**Published:** 2025-02-15

**Authors:** Haider Adnan Alvan, Zohreh Jabbarzadeh, Javad Rezapour Fard, Parviz Noruzi

**Affiliations:** Department of Horticultural Science, Faculty of Agriculture, Urmia University, Urmia, Iran

**Keywords:** Antioxidant activity, Ion leakage, Malondialdehyde, Osmotic stress, Sweet William, Physiology, Plant sciences

## Abstract

Salinity is considered as one of the most important environmental stresses in plant growth and productivity around the world by arid and semi-arid areas; therefore, the development of an efficient strategy against salt stress in crops is urgently needed. Application of Se thus appeared to be an efficient approach for the improvement of plant growth and productivity under saline condition. This study investigated the effects of salinity stress by applying different NaCl levels (0, 30, 60, and 90 mM) in combination with foliar application of Se at different levels (0, 5, 10, and 15 µM) on morpho-physiological and biochemical traits of *Dianthus barbatus*. Done in a factorial design and completely randomized layout with three replications, the findings showed that salinity caused significant reduction in growth, increased electrolyte leakage and malondialdehyde levels, and increased activities of antioxidant enzymes. At an increase in growth defects among the saline treatments, a positive level of 90 mM NaCl was recorded, whereas the imposition of Se improved some growth traits in most aspects: phenolic and flavonoid contents; antioxidant capacity was boosted in Se-stressed plants. Indeed, at 10µM application level in most of salinity treatments and controls, enhancing the salinity tolerance was reflected. These evidences show cell membrane stabilization of Se through maintaining compounds with various protective functions coupled with enhancing their antioxidant enzyme capacity at efficient low doses. In conclusion, Se application through foliage was an effective method to enhance the plant’s tolerance capacity against salinity in sweet william and could turn out to be a sustained solution for agricultural production under salinity conditions.

## Introduction

Sweet william (*Dianthus barbatus* L.), a member of the Caryophyllaceae family, is a widely cultivated ornamental plant in gardens. It can reach a height of up to 90 cm, with upright stems and leaves ranging from 7.5 to 15 cm in length. The flowers, which may appear singly or in clusters, come in a variety of colors, including pink, white, red, purple, and their blends. These flowers are also fragrant, and double-flowered cultivars are particularly popular^1^.

Plant growth and development are constantly challenged by biotic and abiotic stresses^2^. Among the abiotic stresses, namely salinity, drought, and temperature extremes, salinity is considered one of the major threats to sustainable agriculture worldwide and contributes to soil desertification^3^. Saline soils are defined by high salt concentrations (electrical conductivity > 4 ds/m), exchangeable sodium less than 15%, and pH lower than 8.5^4^. Highly soluble chloride and sulfate ions have a great impact on soil salinity, with sodium chloride being the major one^5^. High amounts of sodium ions can destroy cell membranes and change the structure of proteins^6^.

The plants, upon exposure to salt stress, have several biochemical, physiological, morphological, and molecular changes. It exerts a negative effect on plant physiology by hindering the normal activity of the plants due to a reduction in the water-absorbing capacity of the plants caused by osmotic imbalance, leading to dehydration and impeded growth^7^. Accumulation of toxic ions, mainly sodium and chloride, causes nutrient imbalance and negatively affects the major physiological functions like photosynthesis and protein synthesis^8^. Exorbitant levels of salt induce oxidative stress, characterized by the production of ROS that may damage cellular macromolecules^9^. Antioxidative defense system antioxidant comprising of enzymes (like superoxide dismutase, catalase, peroxidase) and non-enzymatic (like ascorbate and glutathione) antioxidants of the plant come into effect, hence bringing into light how to deal with stresses such as drought, waterlogged conditions, extreme temperatures, radiations, chemical contaminations^10^. Pro-longed duration of stress sometimes can affect a big loss because there are potential risks, causing destruction of certain homeostatic capabilities leading to further damages of cells^11^. In addition, salt stress changes the hormonal balance to close the stomata and hence reduces gas exchange, further restricting photosynthesis. These, all together, bring about growth retardation, chlorosis, leaf injury, and loss of yield in crops^12^.

Though not essential, Se has been reported with immense potential in increasing tolerance to salinity stress in plants^13^. It plays a positive role in the mitigation of the adverse effects of salt stress by improving physiological and biochemical responses. Selenium is involved in water uptake and maintenance of osmotic balance through regulating ion transport, hence reducing toxic accumulation of sodium and chloride ions in plant tissues^14^. Moreover, selenium enhances the antioxidant defense system of the plant through increased activity of enzymatic antioxidants such as superoxide dismutase, catalase, and peroxidase, and non-enzymatic antioxidants like ascorbate and glutathione, which collectively reduce oxidative damage due to ROS^15^. Besides, selenium improves chlorophyll content and photosynthetic efficiency, contributing to sustained growth and productivity under saline conditions. It also modulates stress-related hormones, including abscisic acid (ABA), promoting better stomatal regulation and water conservation^16^. Overall, selenium’s protective effects lead to improved plant growth, reduced oxidative stress, and enhanced tolerance to salinity^17^.

Although salinity is an abiotic stress that negatively affects the growth and ornamental value in many plant species, research within the ornamental plants regarding mitigating this, especially with selenium, is scant. The few experiments conducted on farm crops have generally indicated that adding selenium to crops enhances their salt tolerance by correcting physiological functions, intensifying antioxidant phytohormones, and maintaining ion balance. These facts form a background for this experiment, studying the ability of Se to enhance salt tolerance and ornamental values of *Dianthus barbatus* under conditions of NaCl stress. We therefore estimate the basic parameters of the stress resistance: growth response, physiological adaptations, and antioxidant defense mechanisms of the system of detoxification of ROS, along with ornamental traits like fresh and dry weight of flowers. These factors are analysed, where possible, under controlled experimental conditions for new insights in using selenium as a strategy for improving resilience and aesthetic quality of ornamental plants within saline environments. Although the role of Sabrine in the salinity stress mitigation of agronomic crops is well documented, there is little research currently available concerning ornamental plants. Since *Dianthus barbatus* is an important ornamental species and widely cultivated, its selenium experiments under saline conditions are lacking. The present paper bridges this gap by reporting the physiological, biochemical, and morphological performance of Dianthus barbatus against selenium foliar application under NaCl-induced salinity stress. It is uniquely dealing with the potentials of selenium to improve stress tolerance and ornamental quality, providing new insights into sustainable floriculture and offering innovative strategies for the management of abiotic stress in ornamental plants.

## Materials and methods

### Plant materials and greenhouse conditions

For this experiment, F1 seeds of sweet william, *Dianthus barbatus* ‘Carpet Group’, were provided. These seeds were germinated and cultured within plug trays inside a greenhouse at Urmia University. Three months after germination, when the seedlings had fully established, each seedling was planted into a 12 cm diameter by 9 cm height plastic pot filled with the appropriate soil medium. In this phase, all the plants received the same tap water to uniformly satisfy the plants’ water requirement for proper growth.

Before the experimental treatments were started, the water and soil analyses were made to know their background conditions, which are given in Tables 1 and 2 below, respectively. Greenhouse temperature and relative humidity in both growth and experimental phases of the plants have been carefully monitored. Daytime temperature ranges were maintained from 18 to 21 °C; nighttime, between 13 and 16 °C, with the same relative humidity in the 50–60% range. Photoperiodic condition was around 10–12 h/day and daylight.

**Table 1:** Physical and chemical characteristics of the soil used in the study.

K (mg/kg)	*P* (mg/kg)	Organic C (%)	EC (ds/m)	PH	Soil texture
324	24.20	1.31	1.5	7.36	Sandy loamy

**Table 2:** Results of greenhouse water analysis (anions and cations in meq/l).

CaCO_3_ (mg/l)	K^+^	Na^+^	Mg^+ 2^	Ca^+ 2^	Cl^−^	HCO_3_^−^	CO_3_ ^− 2^	PH	EC (ds/m)
421	0.11	1.2	2.8	6.3	4.2	4.3	0.1	6.95	610

### Experimental design and treatments

The experiment was performed as a factorial trial within a completely randomized design, with three repetitions per treatment. The two treatments studied were salinity stress, induced by sodium chloride (NaCl, Sigma-Aldrich), and the foliar application of elemental selenium (Sigma-Aldrich). Salinity stress treatments were imposed at four levels: 0, 30, 60, and 90 mM NaCl, corresponding to electrical conductivities of 0.61, 4.0, 7.57, and 10.5 dS/m, respectively. These concentrations were selected based on the literature showing their effectiveness in inducing stress tolerance in plants^18^. Salinity treatments were applied through irrigation and maintained for a continuous period of 45 days on four-month-old *Dianthus barbatus* plants. To avoid sudden osmotic shock, salinity levels were gradually increased to the target concentrations over three irrigation sessions. Foliar application of selenium was made at four concentrations: 0, 5, 10, and 15 µM. These treatments of selenium were done along with the treatments of salinity stress at every two-week interval using the spray solution. The distilled water for spraying on the control groups had been used for this very reason so that selenium exposure may be zero.

### Morphological characteristics measurements

The plant height was measured as the length from the crown to the top of the plant with the help of a ruler. Fresh weights of flower, stem (two per pot), and root were recorded using a digital balance (METTLER, PJ300). Drying weight samples of flowers, stems, and roots of each plant in the oven at 72 °C for 24 h were weighed afterward.

### Electrolyte leakage determination

The leaf membrane stability was measured as electrolyte leakage (EL) according to the method of Chakrabarty et al.^19^. Leaf disks (0.2 g) were washed in distilled water and incubated in 15 ml of distilled water at 40 °C for 30 min. The initial conductivity (EC1) was read out, and then the leaf disks were kept in the solution at 100 °C for 10 min. The final conductivity (EC2) was measured after cooling to room temperature. Electrolyte leakage (%) was calculated as:

$$\:\text{E}\text{L}=\:(\text{E}\text{C}1/\text{E}\text{C}2)\:\times\:100$$

### Estimation of MDA content

Malondialdehyde content was assayed according to the method of Horst and Cakmak^20^. Fresh leaf samples (0.2 g) were homogenized in 5 ml of 1% trichloroacetic acid (TCA). The homogenate was centrifuged at 8,000 × g for 10 min. A 1 ml aliquot of the supernatant was mixed with 4 ml of a solution containing 20% TCA and 0.5% thiobarbituric acid (TBA). The samples were heated at 95 °C for 30 min and immediately cooled in an ice bath. After centrifugation, the absorbance of the supernatant was measured at 600 nm and 532 nm. The MDA concentration was calculated using the following formula in µmol/g fresh weight:

$$\:\text{M}\text{D}\text{A}\:(\text{m}\text{o}\text{l}/\text{g}\:\text{F}\text{W})\:=\:(\text{A}532\:\--\:\text{A}600\:/\:155)\times\:100$$

Where:

- MDA is the malondialdehyde concentration,
- A532 is the absorbance at 532 nm,
- A600 is the absorbance at 600 nm,
- FW is the fresh weight of the leaf sample.

### Determination of total antixidant capaciy

The total antioxidant capacity was determined by a method inspired by the scavenging of DPPH radicals^21^. An aliquot of 100 µl of alcoholic extract prepared by grinding 0.5 g of leaves in 5 ml of 85% methanol was added to 1900 µl of DPPH solution and incubated for 30 min in the dark. Later, the absorbance was read at 517 nm in a spectrophotometer. The antioxidant capacity (%) was expressed as:

$$\:\text{A}\text{n}\text{t}\text{i}\text{o}\text{x}\text{i}\text{d}\text{a}\text{n}\text{t}\:\text{c}\text{a}\text{p}\text{a}\text{c}\text{i}\text{t}\text{y}\:\left(\text{\%}\right)=\:(\text{A}\text{b}\text{s}.\:\text{b}\text{l}\text{a}\text{n}\text{k}-\:\text{A}\text{b}\text{s}.\:\text{s}\text{a}\text{m}\text{p}\text{l}\text{e}/\text{A}\text{b}\text{s}.\:\text{b}\text{l}\text{a}\text{n}\text{k})\times\:100$$

Where:

- Abs. blank is the absorbance of the control reaction (without the sample),
- Abs. sample is the absorbance of the test compound (with the sample).

### Plant extract preparation for enzyme activity measurement

Plant extracts were prepared for the determination of catalase (CAT), ascorbate peroxidase (APX), and guaiacol peroxidase (GPX) enzymes according to the methods of Kang and Saltveit^22^. Leaf samples (0.5 g) were ground in 10 ml of extraction buffer containing 0.1 M phosphate buffer, pH 7.5, containing 0.5 mM EDTA for CAT and GPX, and 1 mM EDTA for APX. The extract was then centrifuged at 15,000 × g for 20 min at 4 °C. The supernatant was used for the determination of enzyme activities.

#### Catalase (CAT) activity

Catalase activity was assayed as the disappearance of hydrogen peroxide according to the method of Aebi^23^. We used a reaction mixture containing 0.2 ml of 1% H₂O₂, 2.5 ml of 50 mM phosphate buffer, pH 7, and 0.3 ml of plant extract. The decrease in absorbance at 240 nm was followed spectrophotometrically for 1 min using an extinction coefficient of 43.6 mM⁻¹ cm⁻¹. Catalase activity was calculated as:

$$\:unit\:\frac{\mu\:mol\:H2O2\:mg-1\:protein\:}{min}=\:\frac{do\:D/\text{min}\left(slope\right)\times\:vol.\:of\:assay\:}{Extinction\:coefficient\:\left(43.6\right)}$$

#### Guaiacol peroxidase (GPX) activity

The GPX activity was measured as increase in absorbance due to the formation of tetra-guaiacol at 420 nm, according to Upadhyaya^24^. Reaction mixture consisted of 1 ml of 1% guaiacol, 1 ml of 1% H₂O₂, 2.5 ml of 50 mM phosphate buffer (pH 7.5), and 0.1 ml of plant extract; the total volume was made to 3.0 ml. Thus, spectrophotometrically, the increased absorbance at 420 nm due to the formation of tetra-guaiacol was measured for 1 min. GPX activity was expressed as micromoles of tetra-guaiacol formed per minute per milligram of protein using the formula:

$$\:unit\:\frac{\mu\:mol\:guaiacol\:mg-1\:protein\:}{min}=\:\frac{do\:D/\text{min}\left(slope\right)\times\:vol.\:of\:assay\:}{Extinction\:coefficient\:\left(26.6\right)}$$

#### Ascorbate peroxidase (APX) activity

APX activity was assayed by following the decrease in optical density at 290 nm due to ascorbate oxidation according to Nakano and Asada^25^. The reaction mixture contained 50 mM potassium phosphate buffer, pH 7.0, 0.1 mM H₂O₂, 0.1 mM EDTA, 0.5 mM ascorbate, 0.1 ml of enzyme extract, and water to make up the final volume. The decrease in absorbance through 290 nm was measured spectrophotometrically. APX activity was expressed as Micromoles ascorbate oxidized min^[-1^mg^[-1^protein and was calculated by using formula:

$$\:unit\:\frac{\mu\:mol\:ASA\:mg-1protein\:}{min}=\:\frac{do\:D/\text{min}\left(slope\right)\times\:vol.\:of\:assay\:}{Extinction\:coefficient\:\left(2.8\right)}$$

### Plant extract preparation for total phenol and flavonoid content

For the determination of the total phenol and flavonoid content, 0.5 g of fresh leaf tissue was homogenized in 5 ml of 85% methanol. Ultrasonication of the homogenate was given at 20 °C for 30 min to ensure efficient extraction; after this treatment, centrifugation was carried out at 5,000 × g for 15 min. The resulting supernatant was collected for the spectrophotometric quantification of the phenolic compounds and flavonoids according to a previously described method by Wang et al.^26^.

#### Phenol content measurement

For the determination of phenol content, 1 ml of the leaf extract was mixed with 9 ml of distilled water and 1 ml of Folin-Ciocalteu reagent. After a reaction period of 5 min, 10 ml of a 7.5% sodium carbonate solution was added to the mixture. The reaction mixture was then incubated at room temperature for 90 min, and absorbance was measured at 750 nm using a spectrophotometer. According to Marinova et al.^27^ the total phenol content was estimated by a calibration curve with known standards of phenol. Results were expressed in milligrams of gallic acid equivalents (GAE) per gram of fresh weight (mg GAE/g FW).

#### Flavonoid content measurement

Total flavonoid content was determined using the aluminum chloride colorimetric assay described by Chang et al.^28^. A 40 µl aliquot of the leaf extract was mixed with 150 µl of 5% sodium nitrite solution. After a 5-minute incubation, 300 µl of 10% aluminum chloride solution was added and further incubated for 5 min. To this, 1 ml of 1 M NaOH was added. Further, the absorption of the resultant solution was determined at 380 nm using a spectrophotometer. Flavonoids content was measured according to intensity of color and resultant values compared to standard curve drawn using known standard of flavonoid, usually quercetin. Results were expressed as milligrams of quercetin equivalents (QE) per gram of fresh weight (mg QE/g FW).

### Statistical analysis of data

Data analysis was done using SAS software, version 9.2. Tests of the effect of experimental factors and their interaction on the traits analyzed were done through two-way ANOVA. The GLM procedure was used in order to ensure that analysis was robust and accounted for an unbalanced dataset. Means showing significant ANOVA differences were separated using Duncan’s multiple range test at P ≤ 0.05. The assumptions of normality and homogeneity of variances were checked before ANOVA analysis. Heatmap visualization was done in R software, version 4.4.1, using the ‘pheatmap’ package to depict the treatment effects.

## Results and discussion

### Growth indicators

To study the effects of various levels of salinity stress and foliar selenium application on the growth characteristics of *Dianthus barbatus*, plant height and fresh and dry weights of flowers, stems, and roots were measured.

#### Plant height

Salinity at the higher level caused a serious inhibition in plant height by 35% at 90 mM NaCl in comparison to control plants (*P* < 0.05). Such decreases were effectively alleviated by the foliar application of 10 µM selenium, which significantly enhanced the plant height by 29% and 31% at 60 mM and 90 mM NaCl, respectively, compared to the untreated ones under the same conditions (*P* < 0.05) (Table 3). These results clearly indicate the role of selenium in keeping the plants protective against salinity stress for better growth.

**Table 3:** Effect of different levels of salinity stress, induced by NaCl, and selenium application on the growth indicators of *Dianthus barbatus* ‘Carpet Group’. Means within each column followed by different letters are significantly different (*P* < 0.05).

NaCl concentration (mM)	Selenium concentration (µM)	Plant height (cm)	Stem fresh weight (g)	Stem dry weight (g)	Root fresh weight (g)	Root dry weight (g)	Flower fresh weight (g)	Flower dry weight (g)
0	0	18.33^a − d^ ± 0.764668	14.19^b − e^ ± 0.233157	3.5^b − d^ ± 0.046722	5.89^bcd^ ± 0.222975	3.13^bc^ ± 0.058188	1.22^bc^ ± 0.003337	0.12^cde^ ± 0.005780
	5	21.33^a^ ± 1.851225	16.85^ab^ ± 0.407709	4.81^a^ ± 0.145889	6.4^bc^ ± 0.220665	3.76^a^ ± 0.042608	1.38^ab^ ± 0.016686	0.16^abc^ ± 0.002966
	10	20^abc^ ± 0.578035	16.89^ab^ ± 0.136666	4.62^ab^ ± 0.050502	7.93^a^ ± 0.040462	3.9^a^ ± 0.008830	1.55^a^ ± 0.006675	0.20^a^ ± 0.001669
	15	20^abc^ ± 1.001185	15.8^bcd^ ± 0.282371	4.57^ab^ ± 0.063584	7.01^ab^ ± 0.071030	3.6^ab^ ± 0.027317	1.55^a^ ± 0.006675	0.18^ab^ ± 0.0003480
30	0	16.5 ^cde^ ± 1.529336	12.91^cde^ ± 0.125182	3.32^cde^ ± 0.040463	4.21^efg^ ± 0.0371624	2.37^de^ ± 0.026489	1.02^cde^ ± 0.020300	0.08^efg^ ± 0.003337
	5	17^cde^ ± 1.001185	18.5^abc^ ± 0.314303	4.9^a^ ± 0.138849	5.87^bcd^ ± 0.096088	3.87^a^ ± 0.088486	1.36^ab^ ± 0.008830	0.15^bc^ ± 0.002084
	10	20^abc^ ± 0.578035	15.84^bcd^ ± 0.1094201	3.94^abc^ ± 0.023121	6.64^abc^ ± 0.175231	3.61^ab^ ± 0.023121	1.19^bcd^ ± 0.014547	0.14^bcd^ ± 0.002030
	15	20^abc^ ± 0.578035	13.22^de^ ± 0.2151448	3.80^a − d^ ± 0.054634	5.51^b − e^ ± 0.170430	3.83^a^ ± 0.041816	1^cde^ ± 0.005780	0.1^def^ ± 0.002030
60	0	15^def^ ± 1.607814	11.83^e^ ± 0.228817	3.53^b − e^ ± 0.093026	3.47^gh^ ± 0.099673	1.77^fg^ ± 0.016686	0.95^c − f^ ± 0.010421	0.06^fg^ ± 0.0003338
	5	17.33^b − e^ ± 1.607814	15.34^bcd^ ± 0.337264	4.03^abc^ ± 0.097240	4.72^d − g^ ± 0.087536	2.21^ef^ ± 0.067739	1.19^bcd^ ± 0.012033	0.13^cde^ ± 0.002407
	10	21^ab^ ± 0.333728	15.06^bcd^ ± 0.306031	3.58^b − e^ ± 0.076975	5.12^c − f^ ± 0.057899	2.8^cd^ ± 0.060717	1.19^bcd^ ± 0.018581	0.12^cde^ ± 0.001001
	15	18.67^a − d^ ± 5.702755	12.06^de^ ± 0.236524	3.62^b − e^ ± 0.092726	5.1^c − f^ ± 0.073799	2.73^cde^ ± 0.05213	0.75^efg^ ± 0.005780	0.06^fg^ ± 0.002851
90	0	12^f^ ± 1.634929	5.81^f^ ± 0.174563	2.5^f^ ± 0.051809	2.64^h^ ± 0.014547	1.22^g^ ± 0.014547	0.66^fg^ ± 0.011561	0.04^g^ ± 0.000668
	5	15.33^def^ ± 0.667457	6.4^f^ ± 0.221270	2.7^def^ ± 0.067492	3.38^gh^ ± 0.016686	1.33^g^ ± 0.020024	0.83^efg^ ± 0.005780	0.06^fg^ ± 0.000578
	10	17.33^b − e^ ± 1.203275	5.31^f^ ± 0.125448	1.96^f^ ± 0.037162	3.62^gh^ ± 0.029662	1.62^g^ ± 0.017341	0.89^d − g^ ± 0.008830	0.06^fg^ ± 0.000882
	15	14.33^ef^ ± 1.858122	5^f^ ± 0.123615	2.09^f^ ± 0.113615	3.38^gh^ ± 0.078053	1.37^g^ ± 0.015293	0.62^g^ ± 0.010421	0.04^g^ ± 0.000612

#### Stem fresh and dry weight

Salinity stress significantly reduced fresh and dry weight of stems, with 59% and 28.5% decrease at 90 mM NaCl as compared to the control plants (at *P* < 0.05). The exogenous application of 5 µM selenium alleviated such effects, and it enhanced stem fresh weight by 23% and dry weight by 15% (at *P* < 0.05). Conversely, the higher selenium concentrations of 10 and 15 µM failed to exhibit any marked improvement in stem dry weight. These results provide evidence that low concentrations of selenium are efficient to counteract salinity-induced reduction in stem growth (Table 3).

#### Root fresh and dry weight

Salinity stress significantly reduced root fresh and dry weight, with declines exceeding 50% and 61%, respectively, at 90 mM NaCl compared to control plants (*P* < 0.05). Foliar application of selenium (5, 10, and 15 µM) mitigated these effects at 30 and 60 mM NaCl, significantly increasing root fresh and dry weight (*P* < 0.05). However, at 90 mM NaCl, selenium failed to improve root dry weight. These results indicate that selenium enhances root growth under moderate salinity but is less effective under extreme stress conditions (Table 3).

#### Flower fresh and dry weight

Salinity stress drastically reduced flower fresh and dry weight by 47% and 74%, respectively, at 90 mM NaCl as compared with the control at *P* < 0.05. Selenium foliar application at 5 and 10 µM improved such depressing effect of salinity stress. Selenium at 5 µM increased flower fresh weight by 32% compared with the flowers of the salinized plants that did not receive selenium supplementation. This finding indicated partial restoration of flower biomass under salinity by selenium and pointed out the potential for improving flower growth under saline environments with the application of selenium (Table 3).

This research confirms that there is a significant reduction of fresh and dry weights in stems, roots, and flowers under the salinity stress. Like with other abiotic stresses, salt stress suppresses plants’ growth. The magnitude of reduction varies basing on plant species, developmental stages, as well as levels of salt concentration^29^. The stunted growth acts like an adaptation for survival whereby it enables plants to cope with the salt stress^30^. Salt stress can suppress the expression of the key regulatory genes of cell cycle progression, including cyclins and cyclin-dependent kinases, which reduces cell proliferation in the meristem. This inhibition in growth negatively impacts the plant’s ability to absorb water and nutrients efficiently. Some plants immediately respond to salt stress and stop growing, while others keep growing without responding properly and may die under severe stress^31^. *Dianthus barbatus* belongs to the first group because it exhibits a growth reduction without a total growth arrest. Cell shrinkage immediately follows salt stress due to dehydration of the cell. This is temporary, and cell volume returns later, but the rate of cell elongation remains impaired, and to a lesser extent, reduced cell division becomes responsible for poor root and leaf growth rates^32^. Salinity stress also influences shoot growth by causing marked differences in the growth and damage of the salt-stressed plants from the ones not under any stress. Such a response is due to changes in cell-water relations induced by the modification of osmotic potential outside the root, the osmotic effect, impeding the capacity of the plant for low water intake^33^. Moreover, such reduction in growth triggered by salinity may partly be linked to the osmotic stress impeding transport and absorption of water^34^. This can initiate a hormone-mediated series of reactions that limits stomatal opening, reduces assimilation of CO₂, and lowers photosynthetic rates. Diverting energy from growth to the maintenance of salinity homeostasis, along with reduced carbon gain, may contribute further to such growth decline^35^. These mechanisms can explain the observed reduction in plant stem biomass in our study and agree with the previous results for other ornamental plants and cut flowers under salt stress conditions^36^.

Due to this, roots have a greater exposure to high salt concentration, hence are more susceptible to salinity stress. Salinity impairs the osmotic and ionic effects on root functions, such as water absorption and water-use efficiency, leading to a marked reduction in root biomass^37,38^. In our study, fresh and dry weights of roots significantly decreased under the saline conditions in both fresh and dry weights, showing the high susceptibility of *Dianthus barbatus* to the elevated salt concentrations (Table 3). These findings are in agreement with those obtained in studies of other species, such as carnations, where the application of salt stress depresses root elongation and development^39^.

Similarly, salinity stress led to the decline in fresh and dry weights of flowers. This could be justified by ionic toxicity, mainly influenced by the two ions of sodium (Na⁺) and chloride (Cl⁻), disturbing cellular activity or damaging key growth enzymes. Consequently, these will impair cell division and cells’ ability to extend, ending in a lower flower biomass^40^. These results are in agreement with several reports on the adverse effects of salinity on flower number and quality in various ornamental plants^41–43^. More interestingly, our results show that a relatively higher decrease in flower biomass compared to stems and roots, indicating resource allocation to vegetative organs under stress conditions-a phenomenon that needs further studies.

**Fig. 1:**
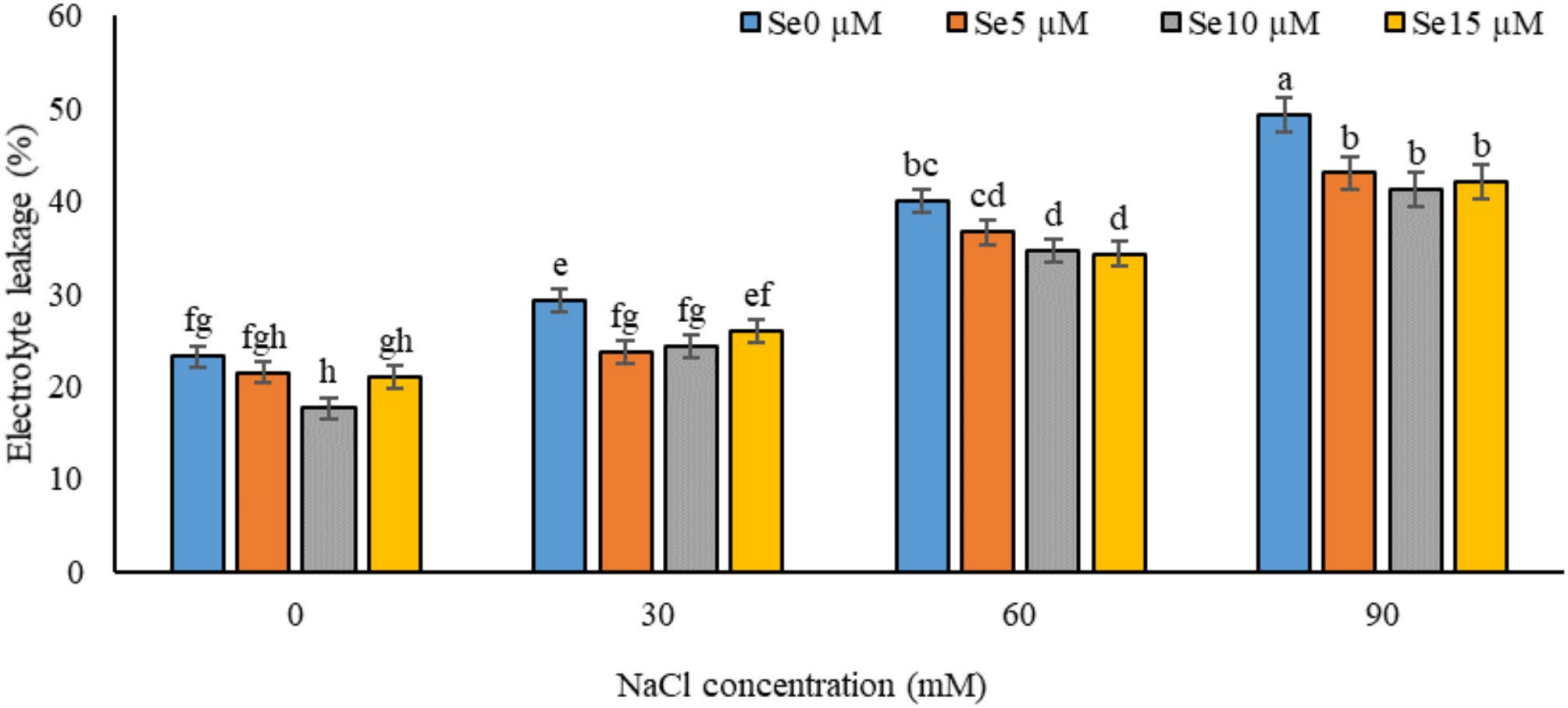
Effect of various salinity stress levels induced by NaCl and selenium application on leaf electrolyte leakage in *Dianthus barbatus* ‘Carpet Group’. (Se: selenium). Error bars represent the standard error of three replicates. Different letters indicate significant differences among treatments (*P* < 0.05).

**Fig. 2:**
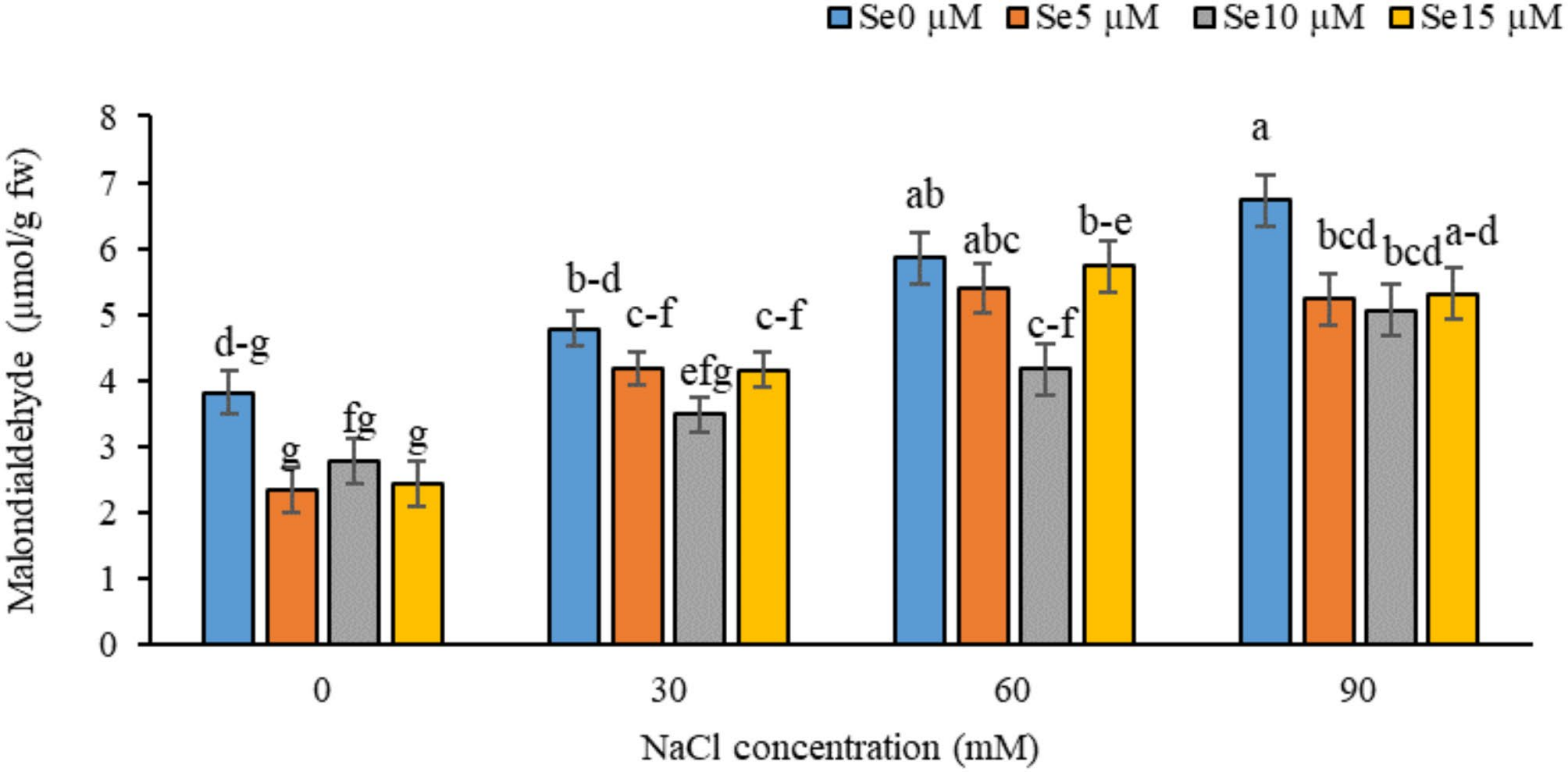
Effect of various salinity stress levels induced by NaCl and selenium application on leaf malondialdehyde in *Dianthus barbatus* ‘Carpet Group’. (Se: selenium). Error bars represent the standard error of three replicates. Different letters indicate significant differences among treatments (*P* < 0.05).

**Fig. 3:**
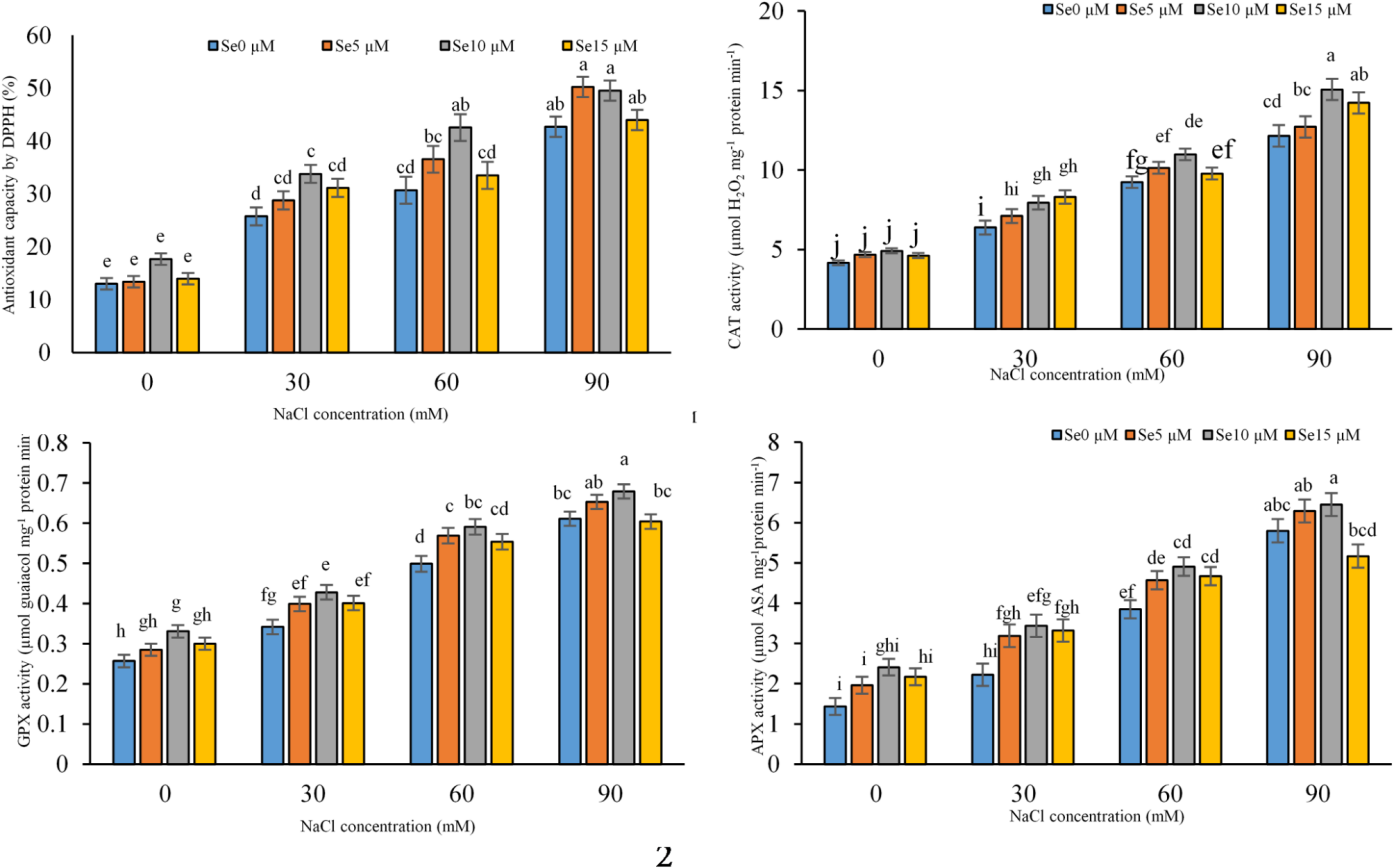
Effect of various salinity stress levels induced by NaCl and selenium application on leaf antioxidant capacity and antioxidant enzymes (Catalase, Guaiacol Peroxidase, and Ascorbate Peroxidase) activity in *Dianthus barbatus* ‘Carpet Group’. (Se: selenium). Error bars represent the standard error of three replicates. Different letters indicate significant differences among treatments (*P* < 0.05).

The study showed that foliar application of selenium greatly diminished the adverse effect of salinity on plant height, fresh, and dry weights of stems, roots, and flowers in *Dianthus barbatus* (Table 3). This shows that selenium plays the most important role in enhancing salinity tolerance probably by way of improving physiological functions impaired under saline conditions. The increase in biomass in the selenium-treated plants can be related to the fact that selenium, through its antioxidant properties, mitigates oxidative damage and cellular structural stabilization. Figures 1, 2 and 3 show evidence that selenium maintains plant growth and vigor despite osmotic and ionic imbalances in the presence of salinity stress. Selenium enhanced growth in plants due to improved photosynthetic efficiency, as manifested by increased activity of photosystem II (PSII). This can be related to selenium’s control over genes responsible for photosynthesis and its regulation of proteins responsible for the encoding of light-harvesting complexes during the process of photosynthesis^44^. Apart from this, there was the hardening of cell membranes due to selenium application that resulted in upholding cell integrity by limiting leakage and electrical conductance of cellular electrolytes (Fig. 1) which supported water and nutrient uptake even in the plant under stress condition^45^. Besides, selenium increases the activity of antioxidant enzymes such as catalase (CAT), ascorbate peroxidase (APX), and guaiacol peroxidase (GPX), which diminish oxidative stress triggered by salinity, thus protecting cellular structures and promoting growth^46^. Selenium also contributes to osmotic regulation within plant cells, improving water uptake and retention, contributing to the alleviation of osmotic stress and biomass accumulation under saline conditions^43^. This is also an important element for the regulation of growth-promoting hormones and nutrient uptake, thus increasing plant stress tolerance^47^. Our results are in agreement with those reported by Hasanuzzaman et al.^48^, who noted that selenium supplementation enhanced biomass accumulation under abiotic stress through enhanced antioxidant activity and reduced oxidative damage. Correspondingly, Farag et al.^49^ demonstrated the role of selenium in counteracting the detrimental effects of salinity on plant growth through cellular homeostasis stabilization and decreased ionic toxicity. Selenium-based compounds have also been reported to increase biomass and enhance photosynthesis in different plant species^44^.

**Fig. 4:**
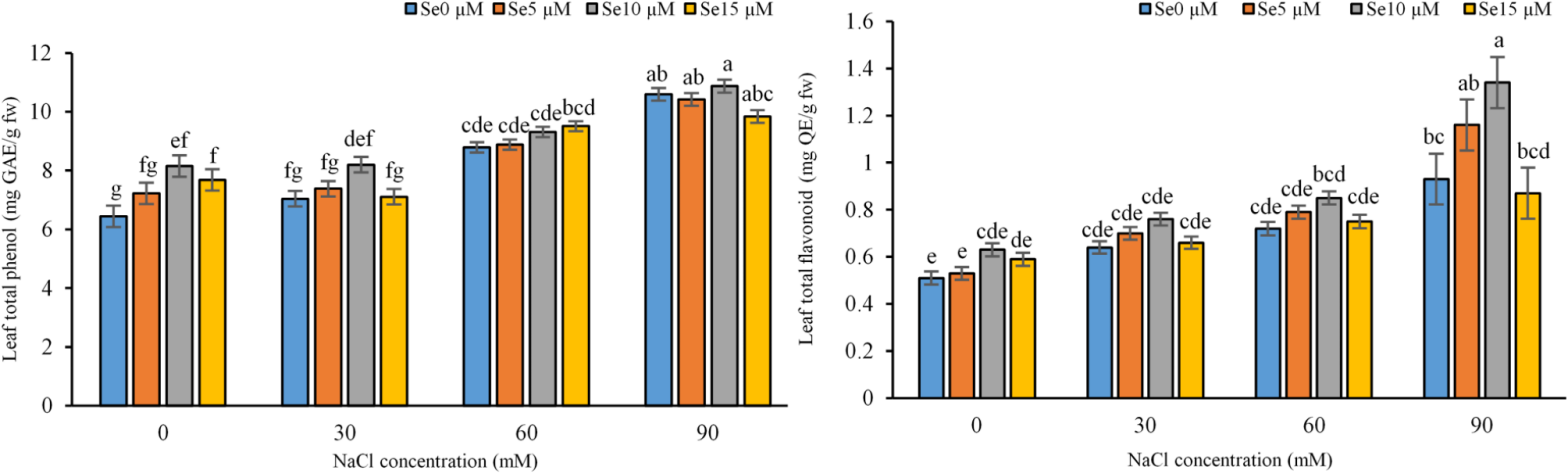
Effect of different levels of salinity stress, induced by NaCl, and selenium application on the leaf total phenol and flavonoid of sweet william ‘Carpet Group’. (Se: selenium) (Error bars represent standard error from three replicates). Different letters indicate that mean values are signifcantly different among samples (*P <* 0.05).

Figure 4 presents the visual effects of salinity stress and selenium application on plant growth. These images show the differences in plant morphology under different treatments, highlighting the positive impact of selenium in mitigating salinity-induced stress.

### The amount of electrolytes leakage (ion leakage)

Salinity stress significantly increased ion leakage in all the tested NaCl concentrations, with maximum ion leakage maintained at 90 mM NaCl. ANOVA, at *P* < 0.05, showed ion leakage to be as high as double that of the control (up to 100%). Such evidence shows that high salinity compromises membrane integrity and makes the membranes more permeable to ion leakage. However, the foliar application of selenium at 5, 10, and 15 µM effectively reduced ion leakage, with a 15% decrease observed at 90 mM NaCl (ANOVA, *P* < 0.05) (Fig. 1). These results indicate that selenium enhances the resistance of plants to salinity stress by improving membrane integrity and reducing electrolyte loss.

It was observed from Fig. 1 that ion leakage from *Dianthus barbatus* leaves significantly rises under the stress of salinity. These increasing leakages of electrolyte might have been caused through several interrelated mechanisms. Salinity causes damage to the cell wall and plasma membrane, leading to the leakage of fluids in the intracellular and vacuolar compartments out into the exterior environment. In turn, such conditions cause increased EC within the solution on account of a high content of electrolytes present in these liquids^50^. Furthermore, salinity stress itself induces the accumulation of H₂O₂, which enhances the peroxidation of lipids against cellular membranes; this increases leakage of ions^51^. Salinity stress also encourages the generation of ROS, themselves capable of causing damage to cellular membranes. As highly reactive compounds, they are able to attack the lipids, proteins, and nucleic acids of membranes-which results in a loss in membrane integrity and increased leakage^52^. Increased ion leakage under salt stress is best explained by the development of oxidative stress from generation and accumulation of ROS. Oxidative stress impairs membrane integrity through peroxidation of lipids and proteins, changes membrane permeability, resulting in leakage of electrolytes and affecting membrane-bound enzyme activities^53,54^. Previous studies have indicated that the ion leakage, under salinity stress, would compromise the maintenance of ionic balance within the plant, which compromises growth and survival^35^.

From Fig. 1, it is clear that the positive effects of the foliar application of selenium on salinity stress in the form of electrolyte leakage are effectively mitigated. Selenium significantly reduced EL in plants under salinity stress, underlining its role in maintaining the integrity and structure of cell membranes. The Se-mediated increase in soluble sugars and free proline improved the photosynthetic efficiency due to the improvement in relative water content and MSI, which minimizes EL and thus decreases ROS production^16^. As shown in Fig. 1, selenium treatment effectively decreased malondialdehyde accumulation under the salinity stress, thereby alleviating the oxidative damage in plant cells. Antioxidative properties of Se have been recorded to prevent lipid peroxidation in cellular membranes under environmental stresses, including salinity stress. Besides, Se strengthens antioxidative systems within the plant cells, which stabilizes cellular membranes against saline conditions, improving ion homeostasis mechanisms^55^. This application assists in maintaining cell ion homeostasis without leakage, which is common under salinity stress conditions through ion leakage^15^. Selenium also maintains osmoregulation and enhances the synthesis of osmoprotectors including proline, soluble sugars, and soluble proteins, which enable maintenance of cellular hydration and reduce excessive electrolyte leakage^16^. In enhancing these protective mechanisms, selenium is effective in countering the effects of salinity stress on ion leakage. Our results are in agreement with other literature that shows supplementation with selenium alleviates the negative effects from salinity stress. For instance, Khan et al.^57^ reported that selenium reduced ion leakage via the regulation of ion homeostasis and hence minimizes oxidative stress. This new knowledge of the inducing role of selenium in selective effects on ion leakage under salinity stress overviews that, besides generally enhancing the stress response of a plant, selenium strengthens cell membrane integrity to reduce ion leakage. These results are therefore important in proving the potential of selenium application as especially helpful in improving tolerance in plants under saline conditions where ionic balance is of most importance to their growth and productivity.

#### Malondialdehyde (MDA) content

Salinity stress significantly increased the MDA level in *Dianthus barbatus* leaves by about 28% at both 60 and 90 mM NaCl compared to the control. The foliar application of selenium, especially at 10 µM, significantly decreased MDA accumulation under salinity stress, showing its protective role (Fig. 2).

This study revealed that salinity stress significantly enhanced the MDA levels, while its increase was ameliorated by selenium supplementation. MDA is one of the major products of lipid peroxidation resulting from oxidative damage to membranes of cells. Salinity can induce oxidative damages in organelles including chloroplast and mitochondria, leading to increased MDA level. This oxidative stress is mainly caused by plasma membrane injury, which triggers the process of lipid peroxidation and hence increases MDA production^58^. Excessive ROS and degradation of unsaturated fatty acids cause cell membrane damage, and hence, lipid peroxidation has been considered one of the major characteristics of cellular injury, and MDA can be used as a reliable marker for oxidative stress^59^. Our findings are in agreement with those reporting higher levels of MDA under salinity stress. For instance, Hasanuzzaman et al.^60^ reported that oxidative stress, due to salinity, induces lipid peroxidation and enhances MDA levels. Atero-Calvo et al.^61^ demonstrated significant increases of MDA correlating with poor growth of the plants and cellular damage.

**Fig. 5:**
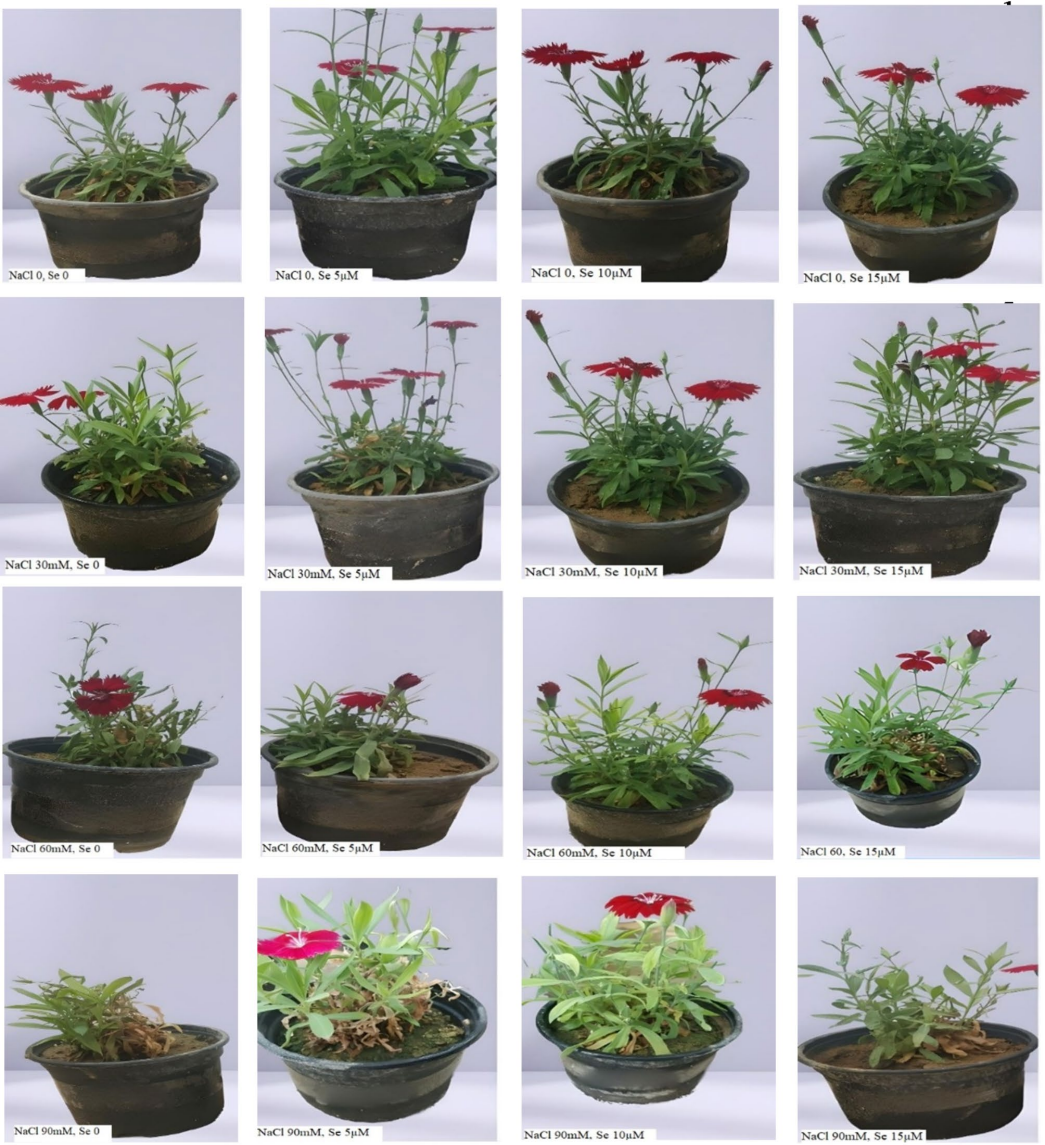
Effect of different levels salinity stress - induced by 0, 30, 60 and 90 mM of NaCl and selenium foliar application − 0, 5, 10 and15µM-on *Dianthus barbatus* ‘Carpet Group’ morphologhy.

When applied at optimum concentrations, selenium greatly promotes plant growth and tolerance to abiotic stresses, including salinity. It ameliorates oxidative stress through a number of mechanisms, which include inhibiting sodium accumulation in plant cells, hence reducing osmotic stress and the chances of oxidative damage^62^. Selenium interferes with pathways involved in the overproduction of ROS, hence minimizing the levels of ROS and the resulting oxidative damage^63^. Moreover, based on the findings of this study, as depicted in Figs. 3 and 5, selenium supplementation improves the enzymatic and non-enzymatic antioxidant defense systems through increased activities of crucial antioxidant enzymes like catalase (CAT), ascorbate peroxidase (APX), and glutathione transferase (GST)^64^. This will help neutralize ROS, decrease oxidative damages, and MDA levels. Selenium protects membrane lipids against oxidative injury through its lowering the contents of superoxide radicals and hydrogen peroxide, avoiding thereby lipid peroxidation and resulting in the decline of MDA accumulation^65^. Selenium application results in decline of MDA production because selenium application promotes antioxidant enzymes that scavenge active oxygen species preventing the oxidation of membrane lipid molecules^66^. Such versatility of selenium in antioxidant defense underlines its potential ability to mitigate oxidative stress, hence improving plant resilience under salinity conditions. These results align with the available literature; for example, Araujo et al.^67^ reported a remarkable decline in MDA levels of stressed plants due to significant induction in activities of antioxidant enzymes superoxide dismutase (SOD) and catalase (CAT) after applying Se. Similarly, Liang et al.^68^ demonstrated that selenium reduced lipid peroxidation and MDA levels in salt-stressed plants, proving its efficiency for the mitigation of oxidative damage induced by salinity.

### Antioxidant properties

#### Antioxidant capacity, Catalase, guaiacol peroxidase, and ascorbate peroxidase activity

Salinity stress significantly increased antioxidant capacity and the activities of catalase, guaiacol peroxidase, and ascorbate peroxidase in *Dianthus barbatus*. Antioxidant capacity was increased by 70% at 90 mM NaCl, which reflected an adaptive response against oxidative damage (ANOVA, *P* < 0.05). Selenium supplementation, especially at 10 µM, further enhanced antioxidant defenses by significantly enhancing the activities of catalase and guaiacol peroxidase under salinity stress, with a notable 66% and 50% increase, respectively. Ascorbate peroxidase activity increased up to 75% at 90 mM NaCl, with 10 µM selenium showing significant effects under stress or control conditions. These results highlight the role of Se in enhancing antioxidant defenses under saline conditions (Fig. 3).

These different levels of ROS produced under salinity stress are balanced by various antioxidant defense mechanisms switched on in plants. Figure 3 depicts this, and it was clearly revealed by the current study that the antioxidant capacity and activity of antioxidant enzymes increase significantly under salinity stress. Salinity causes osmotic stress, inducing water deficit and the accumulation of ROS like superoxide radicals, hydrogen peroxide, hydroxyl radicals, and singlet oxygen. These highly reactive molecules can lead to damage to lipids, proteins, and nucleic acids, including cellular membranes, potentially leading to dysfunction of the cell or cell death^69^. In a normal condition, a balance occurs between ROS generation and scavenging. However, salinity has been known to disturb this equilibrium and result in oxidative stress in plants. This triggers upregulation in antioxidant enzymatic activity such as catalase, ascorbate peroxidase, and glutathione reductase that neutralize ROS and protect cellular components from its oxidative damage^70^. These could be peroxidase enzymes, for example, which break down hydrogen peroxide into water and oxygen, reducing oxidative damage^71^.

Figure 3 summarizes that our data suggest supplementation of selenium enhances the antioxidant capacity and enriches the activity of key antioxidant enzymes involved in the antioxidant defense system, especially the guaiacol peroxidase GPX^72^. Previous studies reported that the application of selenium under salinity stress often enhanced the expression of genes involved in major antioxidant enzymes including superoxide dismutase (SOD), catalase (CAT), ascorbate peroxidase (APX), guaiacol peroxidase (GPX), and peroxidase (POX). This induction increases the capacity of plants to alleviate oxidative stress caused by high levels of ROS. Se enhances the enzymatic activities of SOD, CAT, APX, GPX, and POX by upregulating the antioxidant enzyme genes, which is important for effectively scavenging ROS, reducing oxidative damage, hence improving antioxidant capacity. Such upregulated antioxidant enzymes catalyze the conversion of harmful ROS into less damaging molecules, hence protecting the cellular components against oxidative stress^73^. Further, selenium enhanced the expression not only of antioxidant enzymes-encoding genes but possibly also interfered with signaling pathways that control stress responses, thereby synergically enhancing the antioxidant defense candidacy of plants against salinity stress^74^. In fact, Song et al.^56^ reported that the application of selenium enhanced the activity of antioxidant enzymes by lowering oxidative damage and enhancing ion balance in the salt-stressed plants. In addition, studies explaining how salinity causes an increase in the activities of various antioxidant enzymes, such as SOD, CAT, and POX, supported these findings in support because of the building up of more ROS inside tissues^73,74^. Selenium generally acts as a co-substrate for these antioxidant enzymes: CAT, SOD, GPX-under saline stress. It enhances SOD activity, protecting mitochondria from oxidative damage, and increases GPX and CAT activity, aiding in ROS removal and reducing membrane peroxidation and MDA levels^66^. Selenium application was also reported to improve oxidative stress tolerance and promote plant growth under saline conditions by enhancing antioxidant enzyme activity in research by Luo et al.^75^ and Bandehagh et al.^76^.

### Total phenol and flavonoid content of leaves

Salinity stress significantly increased the content of phenol and flavonoid in the leaves of sweet william in correlation with the severity of salt application. Compared with the control, at 90 mM NaCl a remarkable enhancement of phenol and flavonoid was found at the treatment of 10 µM Se under 90 mM NaCl significantly enhanced the flavonoid content compared to those without selenium treatments, while there was no obvious effect at the other levels of salinity stresses or/and the lower concentration of Se (Fig. 5).

This study showed that salinity stress significantly enhanced the levels of phenolic and flavonoid compounds in the leaf tissues of sweet william (Fig. 5), mainly due to the increased generation of reactive oxygen species (ROS), which may cause cellular damage^77^. In response to oxidative stress, plants activate a complex antioxidant defense system that is comprised of both non-enzymatic components such as phenols and flavonoids, and enzymatic components including catalase (CAT) and peroxidase (POX)^40,78^. Phenols and flavonoids are among the most powerful antioxidants that occur extensively in plant tissues and play a very important role in the protection of cellular structure from ROS-induced oxidative damage^79^ through chelation of metal ions, thereby inhibiting the generation of free radicals^80^. Most of them initiate the shikimic acid or phenylpropanoid pathways under stress conditions^81^, wherein PAL (phenylalanine ammonia-lyase) is an important enzyme in their biosynthesis process^82^. Due to the rich number of hydroxyl groups, flavonoids act as powerful antioxidants that scavenge free radicals, reducing oxidative stress to maintain cellular integrity under abiotic stress^83^. Flavonoids thus play a considerable role in alleviating oxidative damage and maintaining cellular integrity through modulating cellular redox homeostasis^84^. Several studies have reported increased phenolic and flavonoid contents under salinity stress. For example, Pungin^85^ reported increased phenolic content associated with antioxidant activity enhancement, thus improving stress tolerance, while Sarker and Oba^86^ reported increased levels of flavonoids to manage oxidative stress under saline conditions. These findings highlight the participation of phenolics and flavonoids in plant adaptation to salinity stress and indicate that their enhanced production may improve crop resilience and quality in saline-prone areas.

In this study, selenium increased the levels of phenols and flavonoids in *Dianthus barbatus* under salt stress conditions (Fig. 5). The enhancement of these secondary metabolites might be due to the role of selenium in triggering stress-responsive pathways and antioxidant machinery that contribute to plant defense against oxidative damage and overall salinity stress tolerance. The ability of selenium is being modulated to antioxidant defence systemsthrough stimulating activity of typical antioxidant enzymes; the catalase (the ability of shown in Fig. 3.), ascorbate peroxidases, and the guaiacol peroxidase resulted in diminutive rate of ROS formation and thus increased a positive phenomenon of phenolics/flavanols metabolisms^64^. Phenolic biosynthesis was prompted through stimulation from enzymes such as a PAL activity, amongst several promoting production of stressed plant products increases^72^. Also, it maintains redox balance due to the increase of antioxidant capacity while reducing oxidative damage induced by different stresses^66^. Besides, through the chelation action of metal ions, selenium stabilizes antioxidant compounds in the plant that lead to an enhanced defense mechanism for the plant. Indeed, such roles have been verified where selenium improves the content of phenol and flavonoids under salt stress conditions. For instance, Skrypnik et al.^87^ reported that selenium increased those compounds in the stressed plants, caused by oxidative stress; also, Ghanbari et al.^88^ report an increase of flavonoids contents and antioxidant capacity during salinity-stressed plants. The efficacy of this experiment demonstrates how selenium plays a key role in keeping salinity-stressed-induced oxidative stress from significantly causing plant defense vulnerabilities.

### Heatmap analysis

**Fig. 6:**
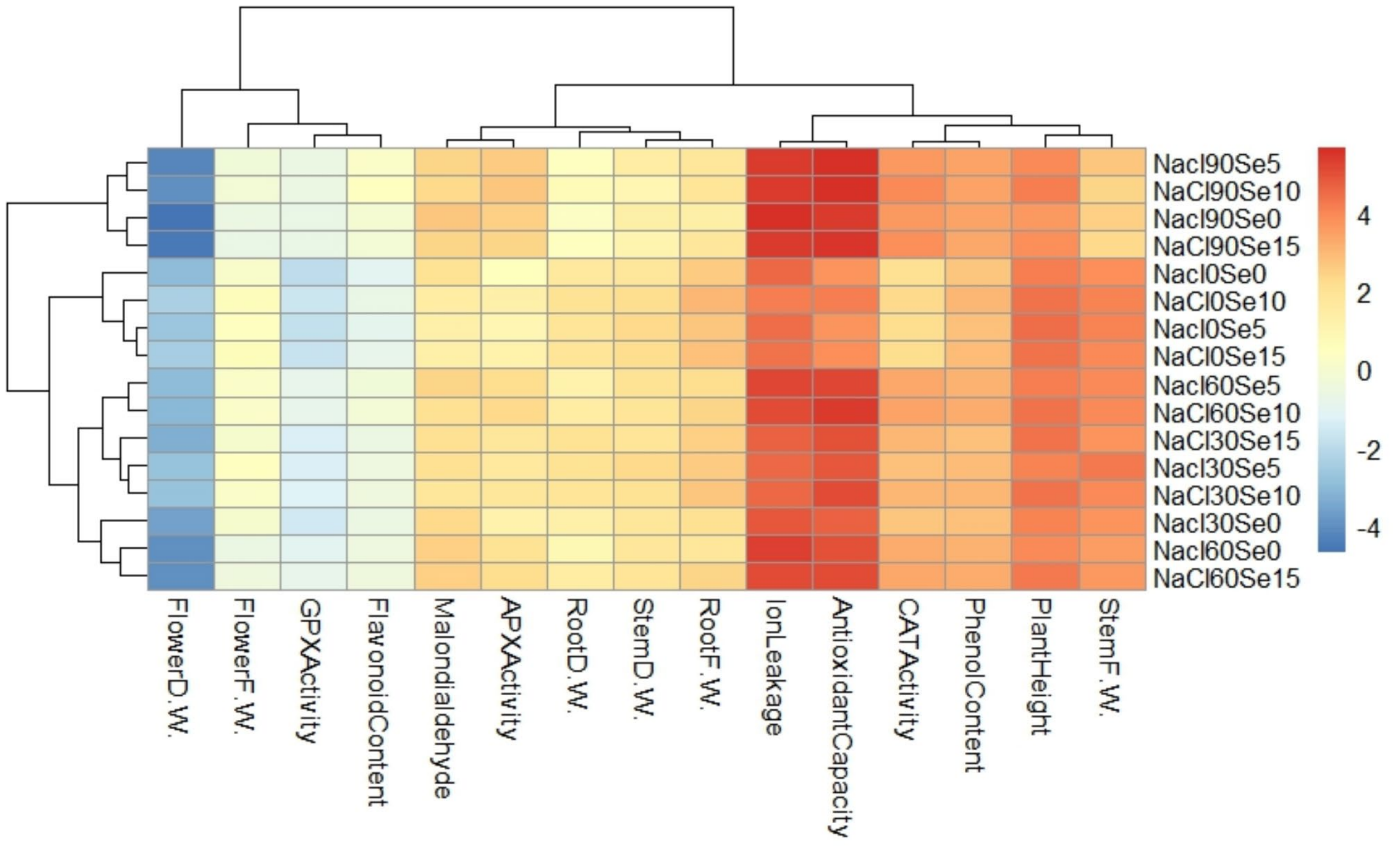
Heatmap clustering of NaCl concentrations (0 [control], 30, 60, and 90 mM) and selenium foliar treatments (0 [control], 5, 10, and 15 µM) based on the morphophysiological and biochemical traits of *Dianthus barbatus* ‘Carpet Group’. The color scale represents standardized mean values, with dark red indicating relatively high mean values and dark blue indicating relatively low mean values.

The heatmap results emphasize the clear amplitude of selenium in mitigating salinity stress induced by NaCl in *Dianthus barbatus* (Fig. 6). High NaCl concentrations increased ion leakage and MDA content, indicative of damage to membranes and oxidative stress. However, under Se application, especially at higher concentrations-for example, NaCl90Se10 and NaCl90Se15-it effectively reduced these negative effects by enhancing membrane stability. Selenium also increased antioxidant capacity and the activity of the major antioxidant enzymes, including CAT, APX, and GPX, which further established its role in the response to oxidative damage. Besides, Se improved some morphological traits lowered by salinity stress, such as fresh and dry weights of roots, stems, and flowers, and plant height. The content of phenolics and flavonoids was higher in Se-treated plants, indicating enhanced antioxidant defense mechanisms. These findings give evidence for the effectiveness of selenium as a mitigating agent of salinity stress, which may act in an overall plant toughening effect, under growth-constraining environmental conditions.

## Conclusion

This study investigates salinity stress on *Dianthus barbatus* and the role of Se foliar application in modulating the ill effects. In this study, we found that increased salinity, especially at 90 mM NaCl, severely hampered plant growth, as reflected by the reduced plant height and lower fresh and dry weights of roots, stems, and flowers. Electrolyte leakage, MDA levels, and antioxidant enzyme activities were enhanced under salinity stress, which pointed to an increase in oxidative stress. Salinity-induced adverse effects were effectively mitigated with Se foliar application, especially at 10 µM. Growth parameters, measured by biomass accumulation, were significantly improved under the treatments in association with reduced electrolyte leakage and MDA levels, thus indicating enhanced membrane stability and reduced oxidative damage. Selenium application also improved phenolic and flavonoid content and enhanced the activities of key antioxidant enzymes, such as ascorbate peroxidase, catalase, and guaiacol peroxidase. These findings have highlighted the important role of selenium in salinity-induced enhancement of plant tolerance. Selenium alleviates the negative impact of salinity on growth and physiological attributes through the enhancement of antioxidant defense systems and reduction in oxidative stress. Our results suggested that selenium foliar application, especially at 10 µM, holds a great promise to be an agronomic practice to enhance sweet william salinity tolerance. Future research is necessary to further optimize selenium application methodologies and its mechanisms underlying plant stress tolerance and productivity enhancement in saline environments.

## Data Availability

All data generated or analysed during this study are included in this published article.

## Declarations

### Research involving plants

The authors confirmed that all methods in regards to plants were carried out in accordance with relevant guidelines in the method section.
